# *NRF2* Gene Polymorphisms, Preconception BMI and Their Interplay in Preeclampsia

**DOI:** 10.3390/ijms27135705

**Published:** 2026-06-24

**Authors:** Ziye Li, Suyan Guo, Xuan Zhou, Junxiang Miao, Fan Xia, Lizhang Chen, Tingting Wang

**Affiliations:** 1 Department of Epidemiology and Health Statistics, Xiangya School of Public Health, Central South University, Changsha 410078, China; liziye_ah@163.com (Z.L.); guosuyan0815@163.com (S.G.); zxabcszjx@csu.edu.cn (X.Z.); miaojunxiang0427@163.com (J.M.);; 2 Hunan Provincial Key Laboratory of Clinical Epidemiology, Xiangya School of Public Health, Central South University, Changsha 410078, China

**Keywords:** preeclampsia, *NRF2*, gene polymorphism, prepregnancy BMI, gene-environment interaction

## Abstract

This study aimed to explore the correlations of nuclear factor erythroid 2-related factor-2 (*NRF2*) gene polymorphisms, prepregnancy body mass index (BMI) and the interaction between them with the risk of preeclampsia (PE). A case–control study was conducted in which pregnant women with PE (*n* = 198) and normotensive pregnant women (*n* = 396) were recruited as the case group and control group, respectively, from two tertiary hospitals in Hunan Province. Data collection was achieved through face-to-face interviews utilizing a standardized questionnaire, along with perinatal health care records. Blood samples were also collected, and genotyping of nine single-nucleotide polymorphisms (SNPs) in the *NRF2* gene was subsequently performed using the MassArray platform. Both univariate and multivariate logistic regression analyses were employed to assess the associations of *NRF2* gene polymorphisms with prepregnancy BMI and their interactions with the risk of PE. Multivariate logistic regression analyses revealed a significant association between prepregnancy BMI and PE susceptibility. Specifically, prepregnancy overweight/obesity (BMI ≥ 24.0 kg/m^2^) was associated with an elevated risk of PE (adjusted *OR* = 4.59, 95% CI: 2.82–7.45), whereas underweight status (BMI < 18.5 kg/m^2^) was correlated with a reduced PE risk (adjusted *OR* = 0.38, 95% CI: 0.18–0.78). The *NRF2* polymorphism rs13005431 exhibited a protective effect against PE under the additive genetic model (adjusted *OR* = 0.59, 95% CI: 0.37–0.93). Furthermore, logistic regression analyses revealed a significant effect of the multiplicative interaction between prepregnancy overweight/obesity and polymorphisms rs35652124 (adjusted *OR* = 0.24, 95% CI: 0.06–0.89) and rs2627765 (adjusted *OR* = 3.62, 95% CI: 1.07–12.23) on susceptibility to PE. These findings collectively underscore the critical and independent roles of prepregnancy BMI, *NRF2* polymorphisms, and their interactions in modulating PE susceptibility, suggesting that the combined effects of metabolic profiles and genetic determinants may act synergistically to shape PE risk.

## 1. Introduction

Preeclampsia (PE) is a multisystem disorder that poses a severe threat to maternal and fetal health. It is characterized by new-onset hypertension after 20 weeks of gestation, accompanied by proteinuria and/or maternal end-organ dysfunction and/or utero-placental insufficiency [1,2]. Globally, PE is estimated to contribute to more than 500,000 preterm births and more than 60,000 maternal deaths annually [3]. In addition, studies have shown that women with PE have a 3- to 5-fold increased risk of severe pregnancy complications (e.g., disseminated intravascular coagulation, pulmonary edema) during pregnancy and a 6-fold elevated risk of hypertension within two years postpartum compared with non-PE pregnancies [4,5]. Notably, despite its profound morbidity, no targeted therapies exist for PE in clinical practice beyond pregnancy termination and symptom management [5]. Thus, prevention and early risk stratification are critical for reducing perinatal morbidity and mortality; elucidating the pathogenesis of PE remains pivotal for achieving this goal [6]. Although multiple hypotheses have been proposed, no consensus has been reached about its etiological mechanisms.

Existing evidence, including the marked ethnic/population disparities in PE incidence and its familial aggregation, suggests that genetic polymorphisms play a central role in PE pathogenesis [7,8]. Nevertheless, the current understanding of PE-associated polymorphisms remains limited, as the currently identified susceptibility loci account for only a minor fraction of its heritable risk. The discovery of novel genetic variants through ongoing research may provide critical insights for PE prevention strategies.

The *NRF2* gene, located on chromosome 2q31.2 and comprising 5 exons, encodes the *NRF2* protein—a master transcriptional regulator of the antioxidant stress response [9]. Given that increased oxidative stress and antioxidant imbalance during pregnancy are established core pathophysiological drivers of PE [10,11,12], *NRF2* is hypothesized to be a key mediator of PE development. This finding is further supported by the significant differences in placental *NRF2* levels observed between PE patients and normotensive pregnant patients [13,14]. Several of the SNPs examined in this study have well-documented biological and clinical effects in other oxidative-stress-related conditions. Among promoter variants, rs6721961 disrupts an ARE-like these loci are linked to earlier onset of Parkinson’s disease and altered risk of sequence, reduces *NRF2* activity, and is associated with acute lung injury [15], peripheral arterial stiffness [16], and most relevantly, early-onset preeclampsia in an Iranian cohort [17]. rs35652124 reduces *NRF2* activity, increases oxidative stress burden, and is linked to alcoholic liver cirrhosis [18], diabetic foot ulcers and type 2 diabetes [19], ischemic stroke [20], and Parkinson’s disease [21]. Among intronic variants, rs13005431 modulates *NRF2* activity and is associated with tuberculosis susceptibility [22]; rs2364723 is linked to tuberculosis risk and reduced FEV1 [23]; rs1806649 is associated with postmenopausal breast cancer and cardiovascular/COPD mortality [24]; and rs7557529, rs6721961, and haplotypes spanning amyotrophic lateral sclerosis [21]. Collectively, these findings indicate that common *NRF2* variants modulate disease risk, predominantly in conditions dominated by oxidative stress and chronic inflammation, both of which are also central to preeclampsia, motivating a systematic evaluation of these variants in PE. However, no study has comprehensively characterized *NRF2* variants in preeclampsia.

In addition to genetic factors, environmental exposures and gene–environment interactions are also implicated in PE etiology. Previous studies have demonstrated that an elevated prepregnancy body mass index (BMI) is associated with increased PE risk, with obesity potentially promoting PE through mechanisms such as insulin resistance, placental hypoxia, and increased oxidative stress [25,26]. Given that both prepregnancy overweight/obesity and *NRF2* dysregulation may converge on oxidative stress pathways within the placenta, we hypothesized that interactions between prepregnancy BMI and *NRF2* polymorphisms could synergistically modulate PE risk. However, this gene–environment interplay remains unexplored.

To address these knowledge gaps, this study aimed to investigate the independent and joint effects of *NRF2* polymorphisms and prepregnancy BMI on PE risk. These findings may advance early risk prediction models and inform personalized interventions targeting oxidative stress pathways in high-risk populations.

## 2. Results

### 2.1. Baseline Characteristics of the Participants

A total of 198 PE patients and 396 normotensive controls were enrolled in this study. The baseline characteristics of the PE patients and controls are shown in Table 1. Significant between-group differences were observed in maternal age, with a greater proportion of participants aged ≥ 35 years in the PE group (*χ*^2^ = 18.417, *p* < 0.001). Education level also differed significantly, as fewer PE patients had received college level or above education (*p* < 0.001). Prepregnancy BMI distributions also showed notable disparities, with higher rates of overweight (27.8% compared with 8.0%) and obesity (12.0% compared with 0.8%) in the PE group (*p* < 0.001). PE patients presented elevated frequencies of hyperlipidemia (*p* = 0.002) and a history of pregnancy complications (*p* = 0.045). Behavioral factors, including passive smoking during the periconceptional period (*p* = 0.003), alcohol consumption during the periconceptional period (*p* = 0.047), and tea intake during the periconceptional period (*p* = 0.004), also showed significant group differences.

### 2.2. Prepregnancy BMI and the Risk of PE

The associations between prepregnancy BMI and PE risk are detailed in Table 2. Multivariable logistic regression analyses were performed to assess the association between prepregnancy BMI and PE risk, with adjustment for potential confounders including maternal age, education level, history of pregnancy complications, hyperlipidemia, colds during the periconceptional period, passive smoking, alcohol consumption, and tea intake during the periconceptional period. Compared with women with a normal prepregnancy BMI, underweight women exhibited a reduced risk of PE development, with an adjusted *OR* (a*OR*) of 0.38 (95% CI: 0.18–0.78). Conversely, overweight/obese women had a significantly elevated PE risk (a*OR* = 4.59, 95% CI: 2.82–7.45). After adjustment for potential confounders, both underweight (a*OR* = 0.38, 95% CI: 0.18–0.78) and overweight/obese status (a*OR* = 5.90, 95% CI: 3.74–9.30) remained significantly associated with altered PE risk.

### 2.3. NRF2 Gene Polymorphisms and the Risk of PE

The associations between *NRF2* gene polymorphisms and the risk of PE are summarized in Table 3. The genotype distributions of rs13005431 differed between the case and control groups (*χ*^2^ = 6.64, *p* = 0.036). Univariate analysis based on genetic models revealed that polymorphisms in rs13005431 were significantly associated with the risk of PE, and this association remained statistically significant after adjusting for potential confounders. Under the primary additive genetic model, the rs13005431 polymorphism demonstrated a significant association with a reduced risk of PE. Each additional C allele was associated with an approximately 41% lower risk (adjusted *OR* = 0.59, 95% CI: 0.37–0.93).

**Figure 1:**
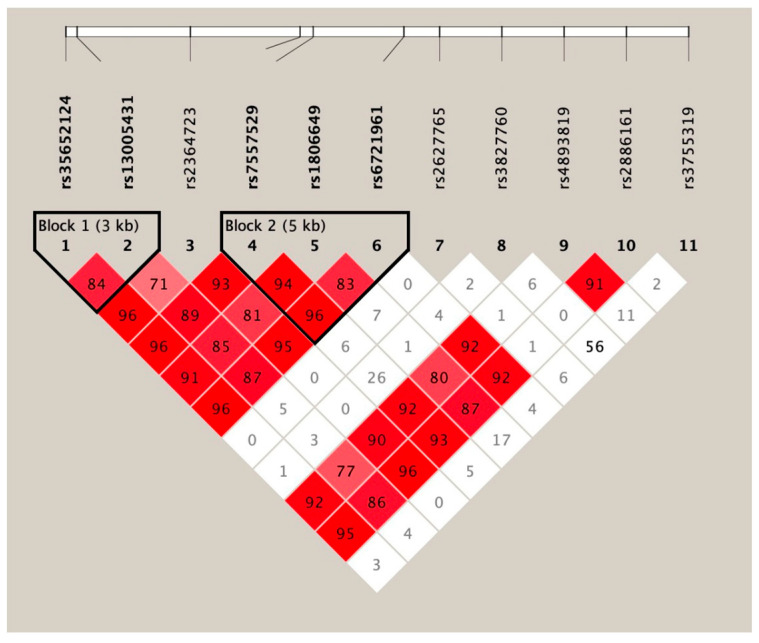
Haplotype analysis of *NRF2* gene.

Haplotype analysis of the *NRF2* gene identified two distinct blocks (Block 1 and Block 2), as illustrated in Figure 1. BLOCK1 (SNP-marker combination rs35652124 and rs13005431) comprised three haplotypes (T-T, C-T, and C-C), whereas BLOCK2 (SNP-marker combination rs7557529, rs1806649, and rs6721961) contained four haplotypes: C-C-T, T-C-G, T-T-T, and T-C-T. As detailed in Table 4, within BLOCK1, the frequency distributions of the C-T (38.4% compared with. 31.7%, *p* = 0.017, *OR* = 1.20) and C-C (7.8% compared with 12.2%, *p* = 0.035, *OR* = 0.68) haplotypes differed significantly between PE patients and controls, suggesting potential associations with PE risk. In contrast, no haplotype in BLOCK2 exhibited significant differences in frequency between groups (all *p* > 0.05).

The color gradient follows the standard Haploview scheme: squares with D′ ≥ 0.7 (values ≥ 70, multiplied by 100 and shown within each square) are colored in red/pink, with darker red indicating stronger LD (higher D′). 

Haplotype blocks (Block 1 and Block 2) are defined by solid black triangles.

### 2.4. Functional Annotation of Key SNPs via eQTL Analysis

To functionally annotate the identified PE-associated variants and investigate their potential regulatory mechanisms, an in silico expression quantitative trait loci (eQTL) analysis was performed. The in silico eQTL analysis revealed that the PE-protective allele (C) of rs13005431 was significantly associated with higher *NRF2* expression (β = 0.084, *p* = 1.68 × 10^−11^; Supplementary Table S4). In contrast, the *rs35652124-C* allele did not show a significant eQTL association (β = −0.022, *p* = 0.086).

### 2.5. Effect of NRF2 Gene Polymorphisms and Prepregnancy Overweight/Obesity Interaction on the Risk of PE

This study comprehensively evaluated the effects of multiplicative interactions between *NRF2* gene polymorphisms and prepregnancy overweight/obesity on PE risk using logistic regression models. The model incorporated *NRF2* SNPs, prepregnancy overweight/obesity, and their interaction terms under a dominant genetic model while adjusting for covariates to control for confounding factors. Analyses revealed significant multiplicative interactions between prepregnancy overweight/obesity and two SNPs: rs35652124 (aOR = 0.24, 95% CI: 0.06–0.89) and rs2627765 (aOR = 3.62, 95% CI: 1.07–12.23) (Table 5). These findings indicate that the combined effects of genetic susceptibility and metabolic status synergistically modulate PE risk.

To further delineate the nature of this interaction, we conducted analyses stratified by prepregnancy BMI status. Stratified analyses by prepregnancy BMI category revealed distinct genetic associations across weight groups (Supplementary Table S5). The protective effect of rs13005431 was only significant in women with normal prepregnancy BMI (aOR = 0.52, *p* = 0.017). In underweight women (BMI < 18.5), two SNPs—rs35652124 (aOR = 0.25, *p* = 0.016) and rs2364723 (aOR = 0.25, *p* = 0.015)—showed strong protective associations not observed in other BMI strata. These findings indicate that prepregnancy BMI modifies the relationship between *NRF2* polymorphisms and PE susceptibility in a stratum-specific manner.

## 3. Discussion

Despite extensive genetic investigations into numerous genes and their SNPs to elucidate their roles in PE pathogenesis, our knowledge of SNPs-PE association remains constrained by limitations in genomic coverage, methodological quality, and statistical power of existing studies. This study is the first to explore the roles of *NRF2* gene polymorphisms, prepregnancy BMI, and their interactions in PE susceptibility. Key findings revealed that specific *NRF2* variants—particularly rs13005431 and the C-C haplotype (rs35652124-rs13005431)—were significantly associated with reduced PE risk, whereas prepregnancy overweight/obesity markedly elevated this risk. Furthermore, multiplicative interactions between pre-pregnancy overweight/obesity and *NRF2* rs35652124 and rs2627765 further modified PE susceptibility, highlighting the interplay between genetic and environmental factors. These results emphasize the multifactorial nature of PE, integrate genetic predispositions and metabolic determinants, and propose *NRF2* variants and BMI as potential biomarkers for early risk stratification and personalized preventive strategies in high-risk pregnancies.

As a master transcriptional regulator of cellular antioxidant responses, *NRF2* is hypothesized to play a key role in the pathogenesis of preeclampsia (PE) by coordinating comprehensive cytoprotective programs—through the induction of genes involved in antioxidant defense, glutathione synthesis, phase II detoxification, and anti-inflammatory processes—to maintain redox homeostasis in the placental microenvironment [5,27,28,29]. However, existing research on *NRF2* and PE has largely focused on its downstream targets or tissue-specific dysregulation. Our study provides novel evidence by linking *NRF2* polymorphisms to PE susceptibility. Specifically, we found that the *NRF2* rs13005431 polymorphism was significantly associated with a reduced risk of PE under an additive model, and in silico eQTL analysis indicated that its protective C allele correlates with higher *NRF2* expression. This enhanced expression likely strengthens placental reactive oxygen species (ROS) scavenging capacity, mitigating oxidative damage and preventing the endothelial dysfunction characteristic of PE, whereas the risk allele (rs13005431 T) may attenuate this protective response, increasing placental vulnerability to oxidative injury [13,30,31]. Furthermore, haplotype analysis in our study revealed that the C(rs35652124)-C(rs13005431) haplotype was associated with a significantly lower PE risk, whereas the C(rs35652124)-T(rs13005431) haplotype was linked to an increased risk. These findings extend the genetic architecture of PE beyond previously established loci (e.g., near *FLT1* and *PGF*), though validation in more diverse populations beyond the Central Chinese cohort studied here remains essential to confirm these associations and elucidate their underlying mechanistic pathways.

Extending these genetic insights, we also examined the interplay between genetic polymorphisms and metabolic status. Consistent with existing evidence, this study confirmed that pre-pregnancy overweight/obesity significantly increases PE risk [32,33,34,35,36,37], which may mediate by its induction of chronic low-grade inflammation and heightened oxidative stress driven by excessive lipid peroxidation and pro-inflammatory adipokines [38,39,40]. Furthermore, we identified significant multiplicative interactions between pre-pregnancy overweight/obesity and specific *NRF2* SNPs (rs35652124 and rs2627765) in modulating PE risk. Specifically, the interaction with rs35652124 exerted a protective effect (adjusted OR = 0.24), whereas the interaction with rs2627765 increased PE risk (adjusted OR = 3.62), indicating that metabolic status can synergistically or antagonistically modulate the effects of *NRF2* genetic variation. Stratified analysis further clarified this context-dependency: the protective effect of rs13005431 was only significant in women with normal pre-pregnancy BMI (adjusted OR = 0.52, *p* = 0.017), while two other SNPs (rs35652124 and rs2364723) showed strong protective associations exclusively in underweight women. We speculate that obesity-induced oxidative stress may overwhelm the *NRF2* pathway (leading to “*NRF2* exhaustion”) or alter its functional state, thereby masking or amplifying the effects of specific genetic variants. Conversely, underweight status might create a distinct biochemical milieu that unveils the protective effects of certain *NRF2* SNPs not observed in normal or overweight/obese groups. These findings underscore the importance of integrating genetic predisposition and metabolic status in PE risk assessment. Therefore, we propose that combining pre-pregnancy weight management with *NRF2* variant screening to optimize PE risk prediction. Future studies should validate these interactions across diverse populations and elucidate their molecular mechanisms to inform targeted preventive strategies.

Several limitations should be acknowledged. First, the case–control design, while useful for exploring associations, is inherently limited by its observational nature. To mitigate potential confounding factors, we conducted a literature review to identify relevant factors, designed a comprehensive questionnaire, and applied multivariable models to adjust for these variables. Second, the data were primarily collected from two tertiary hospitals, excluding lower-level health care facilities, which may limit the sample’s diversity and representativeness, potentially introducing selection bias. Consequently, future studies should validate these findings in broader and more diverse populations. Furthermore, the exclusive recruitment of Han Chinese participants, while methodologically advantageous for minimizing genetic confounding, inherently limits the generalizability of our findings. The allele frequencies of *NRF2* SNPs and their linkage disequilibrium patterns can vary substantially across different ethnic groups and ancestral backgrounds. Therefore, replication studies in other Asian populations, as well as in cohorts of European, African, and Hispanic descent, are essential to determine whether the observed associations and gene-environment interactions are specific to the Han Chinese population or represent universal pathophysiological mechanisms in preeclampsia. Additionally, while this study identified significant genetic associations, the specific biological mechanisms underlying these variants remain unexplored at the cellular and animal model levels. Future research is needed to elucidate these mechanisms and confirm the functional roles of the identified genetic variants.

Despite these limitations, this study provides novel evidence for the synergistic effects of prepregnancy BMI and *NRF2* polymorphisms on PE risk. Clinically, integrating prepregnancy BMI screening and genetic risk assessment (e.g., *NRF2* rs13005431) into prenatal care could enhance risk stratification and support personalized prevention strategies, particularly through intensified oxidative stress monitoring and lifestyle modifications for overweight/obese individuals. Public health initiatives should prioritize prepregnancy weight management education and community-based interventions to mitigate metabolic-related pregnancy risks. Furthermore, future research should validate these findings in multicenter cohorts to strengthen their generalizability.

## 4. Materials and Methods

### 4.1. Study Design and Population

This hospital-based case–control study enrolled participants from Hunan Provincial Maternal and Child Health Hospital and the Third Xiangya Hospital of Central South University in Changsha, Hunan Province, China. Between October 2021 and October 2022, eligible patients with PE were recruited as cases from the obstetrics inpatient population, whereas normotensive pregnant women who received pregnancy care within the same hospitals were recruited as controls. Written informed consent was obtained from both groups, ensuring voluntary participation and full comprehension of the research’s purpose and procedures. Clinical data, questionnaire information, and biological samples were collected, and diagnoses were reviewed through the hospital system.

The study protocol adhered to the ethical principles of the 1964 Declaration of Helsinki and received prior approval from the Ethics Committee of Xiangya School of Public Health, Central South University (No. XYGW-2019-020).

### 4.2. Inclusion and Exclusion Criteria

In this study, PE was diagnosed by obstetricians following the diagnostic criteria outlined in the *Guidelines for the Management of Hypertensive Disorders in Pregnancy* (2020) [41]. According to these guidelines, PE is defined as new-onset hypertension (systolic blood pressure ≥ 140 mmHg and/or diastolic blood pressure ≥ 90 mmHg on at least two occasions ≥ 4 h apart) occurring after 20 weeks of gestation in a previously normotensive woman, accompanied by one or more of the following: proteinuria (≥0.3 g/24 h or protein/creatinine ratio ≥ 0.3 mg/mg), maternal organ dysfunction (renal, hepatic, neurological, or hematological), and uteroplacental insufficiency. To minimize confounding from genetic and cultural heterogeneity, enrollment was restricted to individuals of Han Chinese ethnicity—the predominant ethnic group in Hunan Province, comprising approximately 90% of the local population. This strategy is essential for reducing the risk of spurious associations arising from underlying differences in allele frequencies among distinct ancestral populations. Accordingly, individuals of other ethnic backgrounds, including the Tujia, Miao, Dong, and Yao minority groups native to Hunan, as well as any non-Asian ethnicities, were excluded from the study. Additional inclusion criteria for both cases and controls included: (1) voluntary participation, (2) completion of a standardized questionnaire after providing written informed consent, and (3) provision of adequate biological samples (e.g., venous blood). Exclusion criteria were rigorously applied: (1) multifetal pregnancies, (2) conception via assisted reproductive technologies, (3) chronic medical conditions, including pre-existing hypertension, cardiovascular diseases, or disorders predisposing to secondary hypertension (e.g., hyperadrenalism).

### 4.3. Sample Size Estimation

Sample size estimation was performed for an unmatched case–control study design with a case-to-control ratio of 1:2. Based on the minor allele frequency (MAF) of the studied *NRF2* single-nucleotide polymorphisms (SNPs) retrieved from the dbSNP database, the lowest MAF (0.119 for rs1806649) was used as the expected exposure frequency in the control group (*p*_0_). Assuming a relative risk (RR) of 1.80 derived from previous genetic association studies of PE in Han Chinese populations and setting α = 0.05 (two-tailed) and β = 0.20 (power = 80%) [42], the minimum required sample size was calculated to be 190 cases and 380 controls. The detailed calculation procedure is provided in Supplementary File S1. The present study ultimately enrolled 198 cases and 396 controls, exceeding the estimated minimum sample size.

### 4.4. Sample and Information Collection

The exposures of interest in this study were pre-pregnancy BMI and *NRF2* gene polymorphisms. BMI was calculated as weight in kilograms divided by height in meters squared (kg/m^2^), with both parameters obtained from the perinatal health care handbook provided by local maternal and child health hospitals. BMI categories were defined according to the *Chinese Guidelines for Prevention and Control of Overweight and Obesity in Adults* [43]: underweight (BMI < 18.5 kg/m^2^), normal weight (18.5 ≤ BMI < 24 kg/m^2^), overweight (24 ≤ BMI < 28 kg/m^2^), and obese (BMI ≥ 28 kg/m^2^).

Background information was systematically collected through standardized face-to-face interviews conducted by trained investigators. Data domains included maternal age, education level, annual household income hyperlipemia, history of adverse pregnancies, history of pregnancy complications, as well as active smoking, passive smoking, alcohol exposure, tea exposure, medication history, folate use and colds during the perinatal period. Here, the periconceptional period refers to the time period encompassing three months before pregnancy and the early pregnancy stage. Self-reported disease and medication-related data were cross-validated through a systematic review of perinatal health care records to ensure data accuracy.

For *NRF2* genotyping, peripheral venous blood (3–5 mL) was collected by nurses using EDTA-coated (ethylenediaminetetraacetic acid) anticoagulant tubes within 24 h of hospital admission. Following collection, blood samples were immediately stored at 4 °C and transported to the laboratory under temperature-controlled conditions within 12 h. Samples were centrifuged at 3500 rpm for 15 min to separate plasma and cellular components into two equal aliquots. These aliquots were labeled with anonymized study IDs and stored in −80 °C ultra-low temperature freezers until further analysis.

### 4.5. SNP Selection and Genotyping

Prior to genotyping, single nucleotide polymorphisms (SNPs) in the *NRF2* gene were identified through a systematic search of relevant domestic and international databases, guided by prior studies and cross-referenced with the NCBI database. SNPs with minor allele frequencies (MAFs) below 5% were excluded from subsequent analysis. The MAF distribution of *NRF2* SNPs is detailed in Table S1. Twelve candidate genetic loci were included in this study.

Genomic DNA was extracted using the QIAamp DNA Blood Mini Kit (Qiagen, Valencia, CA, USA) in accordance with the manufacturer’s protocol. Genotyping was performed using matrix-assisted laser desorption/ionization time-of-flight mass spectrometry on the Mass ARRAY system(Agena Bioscience, San Diego, CA, USA). The workflow included: primer design for targeting candidate SNPs (primer sequences are presented in Supplementary Table S3); primer synthesis and dilution; DNA extraction; multiplex PCR amplification; shrimp alkaline phosphatase treatment to dephosphorylate residual nucleotides; single-base extension reactions; resin purification to remove salts; spectrometric data acquisition and analysis. To minimize bias, laboratory personnel conducting genotyping were blinded to case–control status.

### 4.6. Statistical Analysis

A double-entry database was constructed using EpiData 3.1 (EpiData Association, Odense, Denmark), with independent data verification by two researchers to ensure accuracy. Hardy–Weinberg equilibrium (HWE) testing for the 10 *NRF2* SNPs was performed via Haploview v4.2 (Broad Institute, Cambridge, MA, USA), with compliance defined as *p* > 0.05. The genotype distribution of rs13001694 in controls deviated from HWE (*p* < 0.05) (Table S1) and was consequently excluded from downstream analyses.

Categorical variables are presented as counts (percentages). Differences in baseline characteristics were assessed using the chi-square or Fisher’s exact test. Associations of pre-pregnancy BMI and *NRF2* polymorphisms with PE risk were evaluated using logistic regression, yielding odds ratios (ORs) and 95% confidence intervals (CIs). We constructed a multivariate model adjusted for confounders that differed significantly between cases and controls, including maternal age, education level, history of pregnancy complications, hyperlipidemia, and colds, passive smoking, alcohol exposure and tea intake during the periconceptional period. In the genetic analyses, genotypes for each SNP were denoted as homozygous for the major allele (e.g., CC), heterozygous (e.g., CT), and homozygous for the minor allele (e.g., TT). Association testing was performed under the additive model (effect per copy of the minor allele).

Linkage disequilibrium (LD) and haplotype blocks were analyzed using Haploview v4.2 (results in Table S2). Strong LD (r^2^ > 0.8) was observed between rs2364723 and rs2886161, and between rs7557529 and rs4893819; one SNP from each pair (rs2886161 and rs4893819) was excluded from subsequent interaction analyses. Haplotype distributions were compared between cases and controls.

To explore potential functional mechanisms of the identified PE-associated *NRF2* variants, we performed an in silico eQTL analysis. Summary-level data for the association of SNPs rs13005431 and rs35652124 with *NRF2* (ENSG00000116044) expression were retrieved from the IEU Open GWAS project (https://gwas.mrcieu.ac.uk/ (accessed on 18 December 2025)).

To examine gene-environment interactions, we used multivariate logistic regression to test for multiplicative interaction between *NRF2* polymorphisms and pre-pregnancy overweight/obesity on PE risk. Given that underweight status may act as a protective factor with limited relevance to interaction effects, only overweight/obesity was included in this analysis. The model included PE status as the dependent variable, with the SNP, overweight/obesity status, relevant confounders, and their interaction term as independent variables. Statistical significance was set at *p* < 0.05. Based on the modifying effect of prepregnancy body weight suggested by these interaction analyses, we further performed stratified analyses based on prepregnancy BMI category (underweight: <18.5 kg/m^2^; normal weight: 18.5–23.9 kg/m^2^; overweight/obesity: ≥24.0 kg/m^2^). Within each BMI stratum, separate multivariable logistic regression models were applied to assess the associations between *NRF2* SNPs and PE risk, adjusting for the same set of covariates as in the primary analysis.

Statistical analysis was performed using R software, version 3.5.0 (R Foundation for Statistical Computing), and SPSS 26.0 software (SPSS Inc., Chicago, IL, USA), with a two-tailed *p*-value < 0.05 considered statistically significant.

## 5. Conclusions

In summary, this study revealed significant associations of prepregnancy BMI and *NRF2* gene polymorphisms with PE risk. The statistically significant multiplicative interactions observed between prepregnancy overweight/obesity and specific *NRF2* SNPs collectively underscore the critical role of gene–environment interplay in PE pathogenesis. These findings highlight the imperative to integrate genetic predispositions and metabolic determinants for refined risk stratification and support the development of personalized prevention strategies, such as combining preconception weight management with genetic screening. This work provides a foundation for elucidating the underlying molecular mechanisms and developing targeted interventions to mitigate PE risk in high-risk populations.

## Figures and Tables

**Table 1:** Baseline characteristics of PE cases and controls.

Baseline Characteristic	Control Group (*n* = 396)	Case Group (*n* = 198)	*χ* ^2^	*p*
Maternal age			18.417	<0.001
<35	346 (87.4%)	145 (73.2%)		
≥35	50 (12.6%)	53 (26.8%)		
Education level			18.824	<0.001
Less than junior high school	8 (2.0%)	16 (8.1%)		
High school or Technical secondary school	44 (11.1%)	35 (17.7%)		
College or above	344 (86.9%)	147 (74.2%)		
Annual household income (RMB)			1.204	0.558
<60,000	75 (18.9%)	44 (22.3%)		
60,000–100,000	135 (34.1%)	69 (34.8%)		
>100,000	186 (47.0%)	85 (42.9%)		
Pre-pregnancy BMI ^a^ (kg/m^2^)			96.400	<0.001
<18.5	76 (19.2%)	10 (5.1%)		
18.5–23.9	285 (72.0%)	109 (55.1%)		
24.0–27.9	32 (8.0%)	55 (27.8%)		
≥28.0	3 (0.8%)	24 (12.0%)		
Hyperlipemia			9.353	0.002
No	391 (98.7%)	187 (94.4%)		
Yes	5 (1.3%)	11 (5.6%)		
History of adverse pregnancies			2.655	0.103
No	234 (59.1%)	104 (52.5%)		
Yes	162 (40.9%)	94 (47.5%)		
History of pregnancy complications				
No	358 (90.4%)	168 (84.8%)	4.022	0.045
Yes	38 (9.6%)	30 (15.2%)		
Colds during the periconceptional period ^b^			10.284	0.001
No	280 (70.7%)	164 (82.8%)		
Yes	116 (29.3%)	34 (17.2%)		
Active smoking during the periconceptional period ^b^			0.737	0.389
No	390 (98.5%)	193 (97.5%)		
Yes	6 (1.5%)	5 (2.5%)		
Passive smoking during the periconceptional period ^b^			9.134	0.003
No	221 (55.8%)	136 (68.7%)		
Yes	175 (44.2%)	62 (31.3%)		
Alcohol consumption during the periconceptional period ^b^			3.953	0.047
No	371 (93.7%)	193 (97.5%)		
Yes	25 (6.3%)	5 (2.5%)		
Tea intake during the periconceptional period ^b^			8.309	0.004
No	357 (90.2%)	162 (81.8%)		
Yes	39 (9.8%)	36 (18.2%)		
Medication history during the periconceptional period ^b^			3.469	0.063
No	239 (60.4%)	135 (68.2%)		
Yes	157 (39.6%)	63 (31.8%)		
Folic acid supplementation during the periconceptional period ^b^			0.514	0.476
No	7 (1.8%)	2 (1.0%)		
Yes	389 (98.2%)	196 (99.0%)		

^a^: Body Mass Index (classification according to Chinese standard for obesity BMI). ^b^: from 3 months before pregnancy to early pregnancy.

**Table 2:** The association of pre-pregnancy BMI with the risk of PE.

Pre-Pregnancy BMI	Case Group (*n* = 198)	Control Group (*n* = 396)	*OR* (95% CI)	a*OR* (95% CI) ^a^
18.5–23.9	109 (55.1%)	285 (72.0%)	1.00	1.00
<18.5	10 (5.0%)	76 (19.2%)	0.34 (0.17, 0.69)	0.38 (0.18, 0.78)
≥24.0	79 (39.9%)	35 (8.8%)	5.90 (3.74, 9.30)	4.59 (2.82, 7.45)

^a^: Adjusted for maternal age, education level, history of pregnancy complications, hyperlipemia, colds during the periconceptional period, passive smoking during the periconceptional period, alcohol exposure during the periconceptional period, and tea intake during the periconceptional period.

**Table 3:** The association of *NRF2* gene polymorphisms with the risk of PE.

SNP	Case Group (*n* = 198)	Control Group (*n* = 396)	*χ* ^2^	*p*	Multivariate Analysis	
					a*OR* (95% CI) ^a^	*p*
rs35652124						
Genotype			0.31	0.856		
CC	67 (33.8)	137 (34.6)			1.00	-
CT	84 (42.5)	173 (43.7)			1.18 (0.73, 1.92)	0.576
TT	47 (23.7)	86 (21.7)			1.26 (0.72, 2.23)	0.737
Additive model	-	-			0.93 (0.72, 1.19)	0.683
rs13005431						
Genotype			6.64	0.036		
TT	165 (83.3)	300 (75.8)			1.00	-
TC	32 (16.2)	85 (21.4)			0.81 (0.47, 1.38)	0.178
CC	1 (0.5)	11 (2.8)			0.11 (0.09, 0.66)	0.240
Additive model	-	-			0.59 (0.37, 0.93)	0.019
rs2364723						
Genotype			0.73	0.695		
CC	51 (25.8)	115 (29.1)			1.00	-
GC	103 (52)	195 (49.2)			1.18 (0.72, 1.93)	0.333
GG	44 (22.2)	86 (21.7)			1.18 (0.65, 2.15)	0.876
Additive model	-	-			0.90 (0.69, 1.17)	0.578
rs7557529						
Genotype			0.12	0.941		
TT	74 (37.4)	144 (36.4)			1.00	-
TC	94 (47.5)	188 (47.5)			1.04 (0.66, 1.65)	0.705
CC	30 (15.1)	64 (16.1)			0.98 (0.52, 1.87)	0.903
Additive model	-	-			1.01 (0.77, 1.32)	0.754
rs1806649						
Genotype			3.27	0.195		
CC	172 (86.9)	333 (84.1)			1.00	-
CT	26 (13.1)	57 (14.4)			0.81 (0.44, 1.48)	-
TT	0 (0.0)	6 (1.5)			0.00 (0.00, NA) ^b^	-
Additive model	-	-			0.74 (0.45, 1.21)	0.210
rs6721961						
Genotype			1.29	0.524		
GG	99 (50.0)	211 (53.3)			1.00	-
GT	79 (39.9)	155 (39.1)			1.10 (0.71, 1.71)	0.352
TT	20 (10.1)	30 (7.6)			1.41 (0.66, 3.06)	0.708
Additive model	-	-			0.84 (0.63, 1.11)	0.300
rs2627765						
Genotype			2.60	0.272		
GG	141 (71.2)	282 (71.2)			1.00	-
GT	56 (28.3)	105 (26.5)			0.88 (0.55, 1.41)	0.193
TT	1 (0.5)	9 (2.3)			0.21 (0.02, 1.92)	0.177
Additive model	-	-			0.96 (0.66, 1.40)	0.682
rs3827760						
Genotype			1.18	0.554		
AA	178 (89.9)	346 (87.4)			1.00	-
AG	20 (10.1)	49 (12.3)			0.63 (0.32, 1.25)	0.046
GG	0 (0.0)	1 (0.3)			0.00 (0.00, NA) ^b^	-
Additive model	-	-			1.68 (0.86, 3.27)	0.333
rs3775319						
Genotype			2.87	0.239		
AA	90 (45.5)	205 (51.8)			1.00	-
AC	85 (42.9)	158 (39.9)			1.05 (0.68, 1.64)	0.452
CC	23 (11.6)	33 (8.3)			1.52 (0.73, 3.16)	0.380
Additive model	-	-			1.23 (0.93, 1.63)	0.093

^a^: Adjusted for maternal age, education level, history of pregnancy complications, hyperlipemia, colds during the periconceptional period, passive smoking during the periconceptional period, alcohol exposure during the periconceptional period, tea intake during the periconceptional period. ^b^: OR and 95% CI could not be estimated due to zero cell count.

**Table 4:** Haplotypes of *NRF2* gene associated with risk of PE.

Block	Haplotype	Frequency	Case Group	Control Group	*p*	*OR* (95% CI)
Block1 ^a^	T-T	55.5%	53.8%	56.1%	0.560	0.97 (0.76, 1.24)
	C-T	33.6%	38.4%	31.7%	0.017	1.20 (1.15, 1.55)
	C-C	10.9%	7.8%	12.2%	0.035	0.68 (0.45, 0.95)
Block2 ^b^	C-C-T	59.8%	59.7%	59.8%	0.980	1.04 (0.81, 1.33)
	T-C-G	27.6%	30.6%	26.2%	0.105	1.15 (0.88, 1.50)
	T-T-T	7.8%	6.5%	8.5%	0.209	0.75 (0.46, 1.21)
	T-C-T	4.8%	3.2%	5.5%	0.085	0.63 (0.34, 1.20)

^a^ SNP-marker combination rs35652124-rs13005431. ^b^ SNP-marker combination rs7557529-rs1806649-rs6721961.

**Table 5:** Interactions of *NRF2* gene polymorphisms and pre-pregnancy overweight/obesity on PE based on multivariate logistic regression.

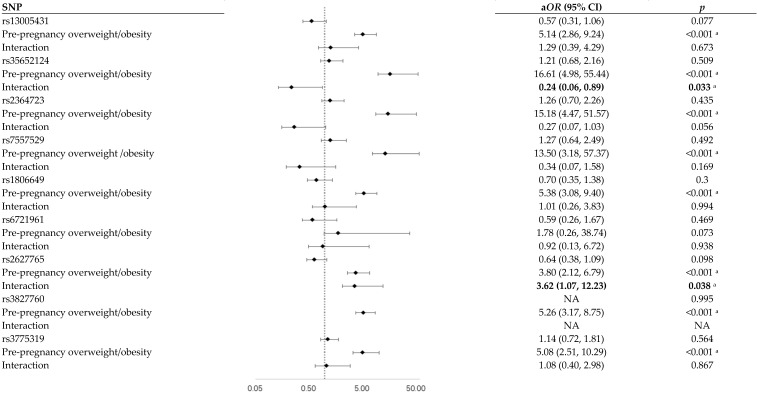

^a^: Adjusted for maternal age, education level, history of pregnancy complications, hyperlipemia, colds during the periconceptional period, passive smoking during the periconceptional period, alcohol exposure during the periconceptional period, tea intake during the periconceptional period.

## Data Availability

The datasets generated and/or analyzed during the current study are available from the corresponding author on reasonable request. Additional supporting data, including minor allele frequencies, primer sequences, and genotypic distribution are provided in the Supplementary Materials (Tables S1–S3).

## Supplementary Materials

The following supporting information can be downloaded at: https://www.mdpi.com/article/10.3390/ijms27135705/s1.
