# Supplementary material for: NRF2 Gene Polymorphisms, Preconception BMI and Their Interplay in Preeclampsia
Source: Int J Mol Sci. 2026 Jun 24;27(13):5705. doi: 10.3390/ijms27135705 (PMC13362413; doi:10.3390/ijms27135705)
Supplement: Supplementary file 1 [file ijms-27-05705-s001.zip › ijms-4206767-supplementary.pdf]

Supplementary File S1. Estimation of sample size.

$$n = \frac{(1+1/c)\times \bar{p} \times \bar{q} \times (Z_\alpha+Z_\beta)^2}{(p_1-p_0)^2} \quad \text{(Formula S1)}$$

$$\bar{q} = 1 - \bar{p} \quad \text{(Formula S2)}$$

$$\bar{p} = \frac{p_1+c\times p_0}{1+c} \quad \text{(Formula S3)}$$

$$p_1 = \frac{p_0\times RR}{1+p_0\times(RR-1)} \quad \text{(Formula S4)}$$

The sample size was calculated according to the formula designed for a 1:c unmatched case-control study (Formula S1), where c is the ratio of the sample size of the case group to the control group (1:2). Z_α and Z_β are the quantiles corresponding to the standard normal distribution of α and β, respectively. In this study, the values of α and β are 0.05 and 0.20, respectively, so $Z_\alpha = 1.96$ and $Z_\beta = 0.84$. The p_0 and p_1 are the estimated exposure rates of the study factors in the control and case groups, respectively. Based on the minimum allele frequency of each SNPs provided in the dbSNP database (Table S1), the lowest value (i.e., 0.119 which is the minimum allele frequency of rs1806649) was used as the expected exposure rate (p_0) of the study factor in the control group. Considering that the incidence of PE is less than 5%, the odds ratio (OR) is approximately equal to the relative risk (RR). Based on the previous study on on the association between gene polymorphisms and the risk of PE conducted in the Han Chinese population [1], a RR value of 1.80 was assumed. Therefore, in this study, the minimum sample size for the case group is 190, and the minimum sample size for the control group is 380.

1. YANAN F, RUI L, XIAOYING L, et al. Association between ACVR2A gene polymorphisms and risk of hypertensive disorders of pregnancy in the northern Chinese population[J]. Placenta, 2020,90: 1-8.

Supplementary Material

Table S1 Genotypic frequencies of maternal *NRF2* polymorphisms and P values of HWE test.

SNPs	Location (GRCH38)	Major Allele	Minor Allele	MAF	Group	Genotype Frequencies[a]			χ^2	*p*
						AA	AB	BB		
rs35652124	2:177265345	C	T	0.476	Control	137 (34.6%)	173 (43.7%)	86 (21.7%)	4.93	0.092
					Case	67 (33.8%)	84 (42.5%)	47 (23.7%)		
rs13001694	2:177254262	A	G	0.148	Control	298 (75.3%)	83 (21.0%)	15 (3.8%)	8.13	0.020
					Case	0 (0.0%)	37 (18.7%)	161 (81.3%)		
rs13005431	2:177256384	T	C	0.148	Control	300 (75.8%)	85 (21.4%)	11 (2.8%)	2.63	0.271
					Case	165 (83.3%)	32 (16.2%)	1 (0.5%)		
rs2364723	2:177261818	C	G	0.481	Control	115 (29.1%)	195 (49.2%)	86 (21.7%)	0.04	0.982
					Case	51 (25.8%)	103 (52.0%)	44 (22.2%)		
rs7557529	2:177270369	T	C	0.581	Control	144 (36.4%)	188 (47.5%)	64 (16.1%)	0.04	0.982
					Case	74 (37.4%)	94 (47.5%)	30 (15.1%)		
rs1806649	2:177253424	C	T	0.119	Control	333 (84.1%)	57 (14.4%)	6 (1.5%)	3.58	0.173
					Case	172 (86.9%)	26 (13.1%)	0 (0.0%)		
rs6721961	2:177265309	G	T	0.733	Control	211 (53.3%)	155 (39.1%)	30 (7.6%)	0.04	0.982
					Case	99 (50.0%)	79 (39.9%)	20 (10.1%)		
rs2627765	2:108897145	G	T	0.124	Control	282 (71.2%)	105 (26.5%)	9 (2.3%)	0.04	0.982
					Case	141 (71.2%)	56 (28.3%)	1 (%0.5)		
rs3827760	2:177266406	A	G	0.905	Control	346 (87.4%)	49 (12.3%)	1 (0.3%)	0.29	0.874
					Case	178 (89.9%)	20 (10.1%)	0 (0.0%)		
rs4893819	2:177266406	T	C	0.581	Control	145(36.6%)	185(46.7%)	66(16.7%)	0.93	0.629
					Case	80(40.3%)	89(45.0%)	29(14.7%)		
rs2886161	2:177263111	C	T	0.481	Control	114(28.8%)	195(49.2%)	87(22.0%)	1.42	0.492
					Case	49(24.7%)	99(50.0%)	50(25.3%)		
rs3755319	2:177265345	A	C	0.381	Control	205 (51.8%)	158 (39.9%)	33 (8.3%)	0.11	0.953
					Case	90 (45.5%)	85 (42.9%)	23 (11.6%)		

[a]: A = homozygous wild—type; AB = heterozygous variant type; BB = homozygous variant type

Table S2 Linkage disequilibrium among candidate locus of *NRF2* gene.

r^2	rs13005431	rs35652124	rs2364723	rs7557529	rs1806649	rs6721961	rs2627765	rs3827760	rs4893819	rs2886161	rs3775319
rs13005431	.	0.1005	0.0661	0.1384	0.4661	0.0331	0.0023	0.0001	0.1043	0.0884	0.0004
rs35652124	.	.	0.7828	0.7670	0.0927	0.4664	<0.0001	<0.0001	0.6926	0.7813	0.0004
rs2364723	.	.	.	0.6445	0.0654	0.3990	<0.0001	<0.0001	0.5929	0.8940	<0.0001
rs7557529	.	.	.	.	0.1195	0.5579	0.0011	0.0029	0.8345	0.6273	0.0008
rs1806649	.	.	.	.	.	0.0236	0.0029	<0.0001	0.0872	0.0719	0.0011
rs6721961	.	.	.	.	.	.	<0.0001	<0.0001	0.5246	0.3613	0.0003
rs2627765	.	.	.	.	.	.	.	0.0002	<0.0001	<0.0001	0.0015
rs3827760	.	.	.	.	.	.	.	.	0.0004	<0.0001	0.0086
rs4893819	.	.	.	.	.	.	.	.	.	0.5820	0.0039
rs2886161	.	.	.	.	.	.	.	.	.	.	0.0002

Table S3 Primer sequences for SNPs of the *NRF2* gene

SNPs	primer sequences
rs35652124	
1st-PCRP	ACGTTGGATGTTTGCCTTTGACGACCTGAG
2nd-PCRP	ACGTTGGATGAGCTCGTGTTCGCAGTCACC
rs6721961	
1st-PCRP	ACGTTGGATGCTTAGGAGAATGGAGACACG
2nd-PCRP	ACGTTGGATGCCTGCCTAGGGGAGATGTG
rs7557529	
1st-PCRP	ACGTTGGATGGGGAAATGAGATTTGCCAAC
2nd-PCRP	ACGTTGGATGCTCAAAGGGCAAAATGAGAG
rs1806649	
1st-PCRP	ACGTTGGATGTCAAATTGCTCCTCTGTGTC
2nd-PCRP	ACGTTGGATGGCGCTAGACATCAGAATTAC
rs13001694	
1st-PCRP	ACGTTGGATGTGCCACAAAGGTAGAGTCTG
2nd-PCRP	ACGTTGGATGATGTTGCTCTGTGTGAAGGG
rs2364723	
1st-PCRP	ACGTTGGATGTTAACCCAGGCTTGAGGAAC
2nd-PCRP	ACGTTGGATGTTCCTCTGTCCTGACTGAAG
rs2627765	
1st-PCRP	ACGTTGGATGGCTGAGAAAGCTTTGTATCC
2nd-PCRP	ACGTTGGATGTGACTCTAGGACTTCAGATG
rs3755319	
1st-PCRP	ACGTTGGATGTTGCTCATCTTTCCCTTTTG
2nd-PCRP	ACGTTGGATGATTCCACTGGCCCAAGATCC
rs3827760	
1st-PCRP	ACGTTGGATGAATCTCATCCCTCTTCAGGC
2nd-PCRP	ACGTTGGATGCAGCTCCACGTACAACTCTG
rs13005431	
1st-PCRP	ACGTTGGATGTGAGGACAATATTGACTGTG
2nd-PCRP	ACGTTGGATGGCAGACAAGAGATTGTCAGG

Supplementary Table S4. Expression quantitative trait locus (eQTL) analysis for key NRF2 SNPs.

SNP	Effect Allele	Other Allele	Beta (Effect Size)	SE	P-value	Gene (Ensembl ID)	Trait (Source)
rs13005431	C	T	0.0844	0.0125	1.68×10^{-11}	ENSG00000116044	NRF2 expression (IEU Open GWAS)
rs35652124	C	T	-0.0215	0.0126	0.086	ENSG00000116044	NRF2 expression (IEU Open GWAS)

Supplementary Table S5. Association between NRF2 polymorphisms and preeclampsia risk, stratified

SNP	Pre-pregnancy BMI	*P*	aOR (95% CI)
rs35652124	18.5-23.9	0.590	1.09（0.79，1.49）
	<18.5	**0.016**	**0.25（0.08，0.77）**
	≥24.0	0.193	0.65（0.34，1.24）
rs13005431	18.5-23.9	**0.017**	**0.52（0.27，0.97）**
	<18.5	0.391	2.04（0.40，10.04）
	≥24.0	0.104	0.44（0.16，1.19）
rs2364723	18.5-23.9	0.665	1.07（0.77，1.51）
	<18.5	**0.015**	**0.25（0.09，0.77）**
	≥24.0	0.205	0.64（0.32，1.28）
rs7557529	18.5-23.9	0.520	1.11（0.79，1.57）
	<18.5	0.185	0.48（0.16，1.41）
	≥24.0	0.501	0.79（0.40，1.55）
rs1806649	18.5-23.9	0.145	0.58（0.28，1.20）
	<18.5	0.381	2.07（0.41，10.59）
	≥24.0	0.399	0.64（0.24，1.77）
rs6721961	18.5-23.9	0.542	0.89（0.62，1.28）
	<18.5	0.318	0.58（0.20，1.68）
	≥24.0	0.537	0.79（0.37，1.65）
rs2627765	18.5-23.9	0.399	0.81（0.50，1.31）
	<18.5	0.339	0.42（0.07，2.50）
	≥24.0	0.424	1.48（0.57，3.87）
rs3827760	18.5-23.9	0.151	1.81（0.80，4.09）
	<18.5	0.624	1.77（0.17，17.72）
	≥24.0	0.172	2.46（0.67，9.01）
rs3775319	18.5-23.9	0.309	1.19（0.84，1.69）
	<18.5	0.439	1.51（0.53，4.30）
	≥24.0	0.467	1.35（0.60，3.03）

[a]: Adjusted for maternal age, education level, history of pregnancy complications, hyperlipemia, colds during the periconceptional period, passive sm oking during the periconceptional period, alcohol exposure during the periconceptional period, and tea intake during the periconceptional period. by prepregnancy overweight/obesity status.
